# A nomogram based on plasma HSP90α and NLR for predicting prognosis in advanced gastric cancer patients treated with PD-1 inhibitors

**DOI:** 10.7150/jca.114975

**Published:** 2025-07-28

**Authors:** Ziting Qu, Yan Zhang, Lili Lu, Xiaowen Qi, Tengteng Zhang, Zhiyan Zhao, Yiyin Zhang, Kangsheng Gu

**Affiliations:** Department of Oncology, the First Affiliated Hospital of Anhui Medical University, Hefei, Anhui Province, China.

**Keywords:** HSP90α, PD-1 inhibitors, Gastric cancer, Efficacy, Prognosis

## Abstract

**Purpose:** To investigate the use of heat shock protein 90alpha (HSP90α) as a marker for prognostic evaluation and efficacy monitoring in patients receiving PD-1 inhibitors treatment for advanced gastric cancer (AGC).

**Methods:** We investigated the value of HSP90α in AGC patients treated with PD-1 inhibitors from a clinical perspective using human plasma samples.

**Results:** In summary, plasma HSP90α was significantly associated with neutrophil-to-lymphocyte (NLR) in AGC patients at baseline. Regarding short-term efficacy, HSP90α levels decreased considerably after PD-1 inhibitor treatment in the partial response (PR) group (P=0.016). Furthermore, there was no significant difference between HSP90α levels in stable disease (SD) group before and after immunotherapy (P=0.659). However, HSP90α levels were considerably greater in AGC patients at disease progression and eventual PD-1 inhibitor therapy failure compared to baseline (P=0.041, P=0.005). Notably, plasma HSP90α, treatment lines, metastatic sites, and NLR level were independent predictive variables for overall survival (OS) in AGC patients receiving PD-1 inhibitors treatment before and after propensity score matching. Additionally, we constructed the nomogram model depending on the above independent prognostic variables, which can well differentiate the clinical prognosis of patients (P<0.001). And ROC curves, calibration curves, and decision curve analysis curves revealed promising discrimination and accuracy of the nomogram. Finally, plasma HSP90α showed specific prognostic value in different subgroups of clinical characteristics.

**Conclusion:** Plasma HSP90α can be used as a marker for efficacy monitoring and prognostic assessment in AGC patients receiving PD-1 inhibitors treatment. We combined plasma HSP90α, NLR, and clinical characteristics to construct a nomogram for predicting the prognosis of gastric cancer immunotherapy, providing a powerful tool for clinical decision-making.

## Introduction

Gastric cancer (GC) is one of the most prevalent malignant tumours worldwide, and many patients are diagnosed in advanced stages with poor prognosis 1. Over the past decades, the prognosis of advanced gastric cancer (AGC) patients has dramatically improved thanks to the continuous development of comprehensive treatments such as chemotherapy 2, targeted therapy 3, and immunotherapy 4. In particular, immunotherapy is now a standard option for first-line treatment of advanced gastric cancer. However, there is a lack of clinical markers to predict the prognosis of immunotherapy 5. Previous researches have suggested that AGC patients with high programmed cell death ligand-1 (PD-L1) expression, microsatellite instability-high (MSI-H) status, and high tumor mutational burden (TMB) may be a population that benefits from immune checkpoint inhibitors (ICIs) therapy 4,6,7. However, survival advantages of programmed cell death-1 (PD-1) inhibitors in patients with low PD-L1 expression, microsatellite instability-low (MSI-L) / microsatellite stability (MSS) status, and low TMB remain controversial 8. In addition, all the above biomarkers rely on tumour tissue for testing, which is expensive, has a low positive rate, and is difficult to obtain repeatedly for monitoring efficacy. Therefore, finding a biomarker in peripheral blood that is relatively simple to detect, highly accurate, and reproducible is particularly important.

HSP90, a member of heat shock proteins, is a highly conserved molecular chaperone crucial for the maturation of newly generated polypeptides 9. Due to its unique impact on particular cancer types, HSP90 is currently receiving much interest 10. It is generally known that HSP90, specifically HSP90α, is secreted by cancer cells and that its release promotes cancer cell survival, migration, invasion, and stemness via autocrine pathways 11. Previous studies have reported that HSP90α protein expression in tumour tissues is a prognostic parameter in GC patients. And stage, lymph node metastasis, and depth invasion were all substantially correlated with HSP90α expression 12,13. However, the clinical value of HSP90α in plasma is controversial.

Plasma HSP90α has shown favourable value in diagnosing disease, monitoring recurrence, and evaluating prognosis in breast cancer 14, ovarian cancer 15, and lung cancer 16. Another research showed that plasma HSP90α was significantly higher in colorectal cancer and linked with immune cells. However, it failed to show the diagnostic and prognostic value for progression-free survival (PFS) 17. Although plasma HSP90α has good diagnostic capability in GC patients, it does not seem to be a predictor of PFS in GC patients. 18. In terms of immunotherapy, HSP90α was reported to be a potential prognostic biomarker for ICIs combination therapy in hepatocellular carcinoma recently. In addition, high plasma HSP90α was an adverse independent prognostic predictor in lung cancer patients accepting PD-1/PD-L1 inhibitors combination chemotherapy 19. Besides, numerous HSP90 inhibitors with different mechanisms of action have been developed for clinical evaluation with promising results 9,20. However, the role of plasma HSP90α in PD-1 inhibitors treatment, especially in GC, remains unknown. NLR is a systemic inflammatory marker that reflects the inflammatory burden of the tumor. Recent studies have reported that NLR is an independent prognostic factor for AGC receiving PD-1 inhibitors.

This research sought to demonstrate the value of plasma HSP90α in the efficacy monitoring and prognostic evaluation of AGC treated with PD-1 inhibitors. And a nomogram was constructed using HSP90α in combination with multiple indicators to predict the prognosis of GC patients receiving PD-1 inhibitors. Concretely, our objectives are as follows: 1) to describe the correlation between plasma HSP90α and clinicopathologic characteristics before and after propensity score matching (PSM); 2) to explore the relationship between dynamic changes in plasma HSP90α before and after PD-1 inhibitor treatment and short-term efficacy; 3) to explore the value of plasma HSP90α in the prognostic evaluation of overall survival (OS) in AGC patients treated with PD-1 inhibitors; 4) to construct and validate the nomogram model for AGC patients based on independent prognostic factors.

## Materials and Methods

### Patients and data gathering

Data on advanced gastric adenocarcinoma patients treated with PD-1 inhibitors admitted to medical oncology and related departments of the First Affiliated Hospital of Anhui Medical University between September 6, 2018 to December 31, 2022, were retrospectively included in the research. And the inclusion criteria were: (a) histologically confirmed gastric adenocarcinoma; (b) stage IV or unresectable cancer; (c) complete clinical data; (d) accepting at least one course of PD-1 inhibitors treatment. The exclusion criteria were: (a) not scheduled for treatment or evaluation; (b) clinical and laboratory data incomplete; (c) no evaluable target lesions or conversion therapy; (d) patients with acute infectious diseases, cardiovascular disease, or rheumatic diseases when blood collection; (e) with other malignant tumours within 5 years. Ultimately, 130 AGC patients receiving PD-1 inhibitors treatment were enrolled. The First Affiliated Hospital Ethics Committee of Anhui Medical University approved the project (Quick-PJ2022-03-34). Clinical data was gathered from the electronic clinical record system, including gender, age, Eastern Cooperative Oncology Group Performance Status (ECOG PS), metastasis to the peritoneum, liver, lung, lymph nodes, number of metastasis sites, treatment lines, HER-2 status, NLR (absolute neutrophil counts / absolute lymphocyte counts), and plasma HSP90α level. PD-1 inhibitors (sintilimab, nivolumab, etc.) were administered to patients at standard dosages (200 mg Q3W or 3 mg/kg Q2W, etc.). The chemotherapy regimen primarily includes: XELOX (days 1-14: twice daily capecitabine 1,000 mg/m^2^ for each cycle + day 1 intravenous oxaliplatin 130 mg/m^2^) and SOX (days 1-14: twice daily S-1 40-60 mg + day 1 oxaliplatin 130 mg/m^2^). Trastuzumab targeted treatment and Apatinib anti-angiogenic therapy are the two main types of targeted therpy. Based on the patient's clinical situation and preferences, the therapy was chosen.

### Follow up

Follow-up data was gathered over the phone or using an electronic clinical record system. Clinical examination and imaging were carried out every two cycles or whenever there was a discernible decline in clinical symptoms, in accordance with Response Evaluation Criteria in Solid Tumours (RECIST) version 1.1. We evaluated clinical effectiveness using the RECIST version 1.1, which was classified as either progressive disease (PD), stable disease (SD), partial response (PR), or complete response (CR). CR plus PR was the formula used to get the objective response rate (ORR). CR, PR, and SD were used to compute the disease control rate (DCR). OS, which was determined from the initial PD-1 inhibitor medication until death or the last follow-up, served as the study's primary endpoint.

### Measurement of plasma HSP90α

Blood samples were taken from all patients on an empty stomach using EDTA anticoagulation tubes. Samples were centrifuged for ten minutes at 3000 rpm. After being transferred to polypropylene tubes, the plasma supernatant underwent another centrifugation (3000 rpm for 10 minutes). The kit's 96-well plate (Protgen Ltd, Yantai, China) was pre-incubated for 30 minutes at 37 °C in preparation for ELISA analysis. The microtiter plate was first filled with standards (50 μL) and diluted samples (50 μL), followed by anti-HSP90α-HRP conjugated antibody (50 μL), and incubated for one hour at 37°C. Following plate washing, 50 μL of Color Developing Solution A and B were added to each microtiter well. The plates were then incubated with termination buffer at 37°C for 20 minutes to stop the reaction. Lastly, the optical density at 450 nm was measured with a spectrophotometer. The protein content may be calculated based on the standard curve of optical density measurements. Plasma HSP90α levels were collected from AGC patients within one month prior to initial treatment, at the time of short-term efficacy assessment, and at the time of disease progression. An HSP90α value of more than 82.00ng/ml is regarded as abnormal in our laboratory, where the normal value range of HSP90α is 0-82.00ng/ml.

### Statistical analysis

The χ^2^ test or Fisher's exact test was utilized to qualitative variables. Additionally, the paired t-test was applied to analyse quantitative variables. The 1: 1 before and after PSM was performed to reduce the impact of unbalanced baseline clinical characteristics on outcomes. The gender, age, ECOG PS, peritoneum metastasis, liver metastasis, lung metastasis, lymph node metastasis, metastasis sites, treatment lines, HER-2 status, and NLR were enrolled to calculate the individual propensity score. And the caliper value was set to 0.03. Cox regression was used to assess the characteristics. Additionally, stepwise regression was used to create the multivariate Cox model, which included variables with P<0.1 in the univariate Cox analysis. Lastly, the nomogram was constructed using characteristics that had a P<0.05 in multivariate Cox analysis. Receiver operating characteristic (ROC) curves and calibration curves were used to evaluate the model's performance. And the net benefit threshold of prediction was also ascertained by doing a decision curve analysis (DCA). Patients were divided into two subgroups: high-risk and low-risk, based on the median prognostic risk score. The variations in OS across risk strata were compared using the Kaplan-Meier curves and the Log-rank test. R software 4.2.2 along with MSTATA software and SPSS version 26.0 were used for all statistical analyses. A two-sided P<0.05 was considered statistically significant.

## Results

### Association between characteristics and HSP90α

**Figure 1:**
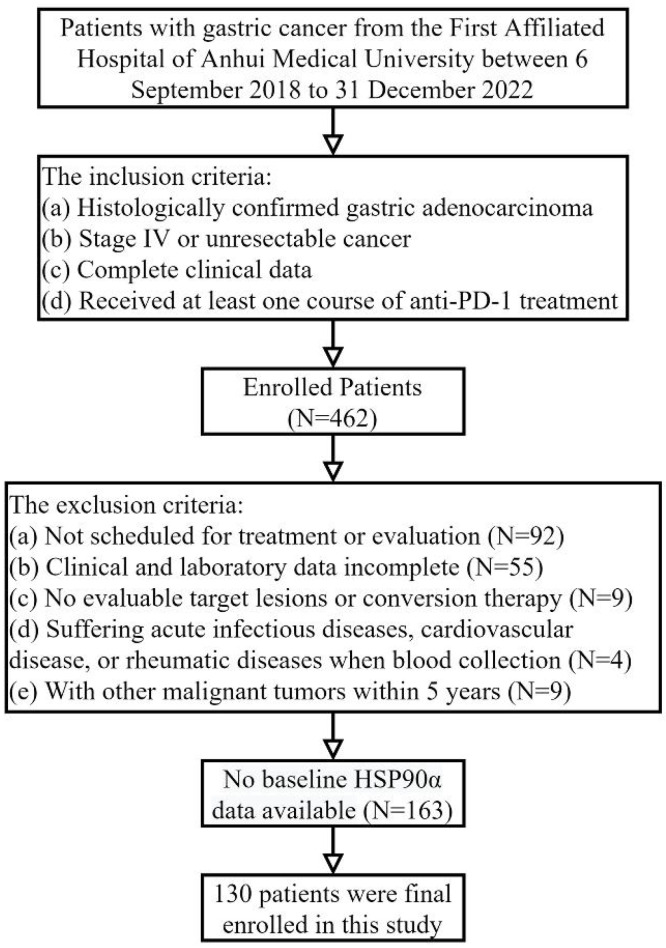
The flowchart of patient selection including inclusion and exclusion criteria.

Table 1 provides patients' baseline characteristics. In this study, 130 AGC patients treated with PD-1 inhibitors were included (Figure 1), including 99 males (76.2%) and 31 females (23.8%). There were 46 patients (35.4%) who were older than 65 and 84 (64.6%) who were younger. 98 patients (75.4%) accepted PD-1 inhibitors in the first-line or second-line, and 32 (24.6%) received third-line or posterior treatment. And 83 OS events occurred, of which 60 were on first- or second-line therapy and 23 were on third- or posterior-line therapy. The PD-L1 test was conducted on 29 of the 130 patients. 11 of these patients had PD-L1 CPS≥1 or deficient mismatch repair (dMMR) status, while the remaining patients had either PD-L1 negative or positive mismatch repair (pMMR) status. Furthermore, 78 patients received PD-1 inhibitor combination chemotherapy, 37 patients received PD-1 inhibitor combined chemotherapy and targeted therapy, and 15 patients received other treatments based on PD-1 inhibitors.

Based on the laboratory reference values for HSP90α levels, the 130 AGC patients were categorized into a high HSP90α group of 79 patients (HSP90α>82ng/ml) and a low HSP90α group of 51 patients (HSP90α≤82ng/ml). And the NLR cutoff value was set at 3, based on widely accepted criteria from previous studies 21. Before PSM, patients with high HSP90α had a higher likelihood of having high NLR (P=0.021) than those with low HSP90α. Other factors such as gender, age, ECOG PS, peritoneum metastasis, liver metastasis, lung metastasis, lymph node metastasis, metastasis sites, treatment lines, and HER-2 status were not significantly different (P>0.05; Table 1). After PSM, the clinical characteristics of 82 patients were more balanced and possible selection biases were reduced, with no significant differences in NLR and other clinical features (P>0.05; Table 1).

### Relationship between dynamic changes in HSP90α and short-term efficacy

**Figure 2:**
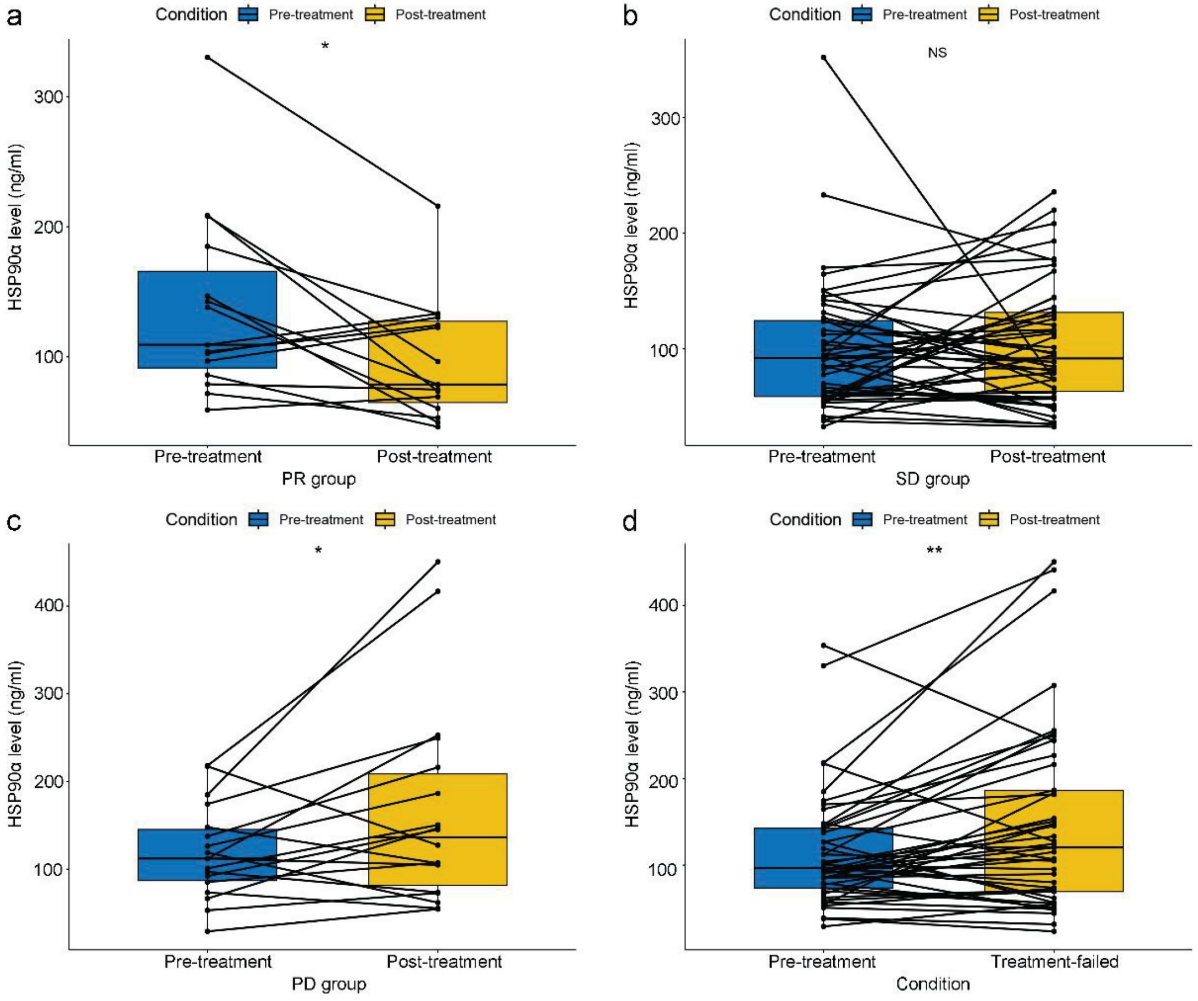
Relationship between dynamic changes in plasma HSP90α and short-term efficacy in AGC patients receiving PD-1 inhibitors treatment in the PR group (A), SD group (B), PD group (C) and the failure of PD-1 inhibitors treatment group (D) (*P < 0.05, **P < 0.01).

In terms of short-term efficacy, no patient attained CR, 30 patients had PR, 30 patients had PD, and 70 patients remained SD. Besides, the ORR and DCR were 23.1% and 76.9%. And data on HSP90α were collected in 15, 46, and 18 cases when PR, SD, and PD efficacy were achieved, respectively. HSP90α was detected in 45 patients when PD-1 inhibitor therapy failed. Specifically, In the PR group, there was a notable drop in HSP90α after PD-1 inhibitors therapy (P=0.016, Figure 2A). In contrast, HSP90α levels did not significantly rise or fall before or after immunotherapy in the SD group (P=0.659, Figure 2B). However, HSP90α levels were significantly elevated when the disease progressed (P=0.041, Figure 2C). In total, after 45 patients' therapy with PD-1 inhibitors failed, HSP90α was measured. Similarly to the PD group, statistical elevations in HSP90α (P=0.005, Figure 2D) were found when PD-1 inhibitors therapy failed compared to baseline in AGC patients.

### Prognostic value of HSP90α in AGC patients before and after PSM

In the present research, the median OS (mOS) of 130 AGC patients was 13.0 (95% CI 11.0-14.9) months, 10.9 (95%CI 7.8-14.0) months in the high HSP90α group, and 17.8 (95%CI 9.1-26.6) months in the low HSP90α group. And the median follow-up was 21.4 (95%CI 19.7-23.1) months. Among 130 AGC patients receiving PD-1 inhibitors treatment before PSM, patients with baseline HSP90α>82ng/ml had shorter OS than those with HSP90α≤82ng/ml (HR: 2.229, 95% CI: 1.370-3.627, P=0.001, Table 2). Additionally, according to Cox regression analysis, metastasis sites (HR: 2.262, 95% CI: 1.327-3.856, P=0.003), treatment lines (HR: 2.158, 95% CI: 1.290-3.610, P=0.003) and NLR (HR: 2.159, 95% CI: 1.383-3.369, P=0.001) were independent prognostic predictors predicting OS in AGC patients (Table 2). After PSM, HSP90α was still an independent prognostic predictor for OS in AGC patients (HR: 1.977, 95% CI: 1.099-3.559, P=0.023; Table 3). Apart from that, Cox regression analyses demonstrated that metastatic sites, treatment lines, and NLR were independent prognostic predictors influencing OS in patients with AGC (HR: 2.978, 95% CI: 1.587-5.587, P=0.001; HR: 2.010, 95% CI: 1.019-3.963, P=0.044; HR: 1.888, 95% CI: 1.061-3.358, P=0.031; Table 3), respectively.

### Establishment and validation of nomogram model

**Figure 3:**
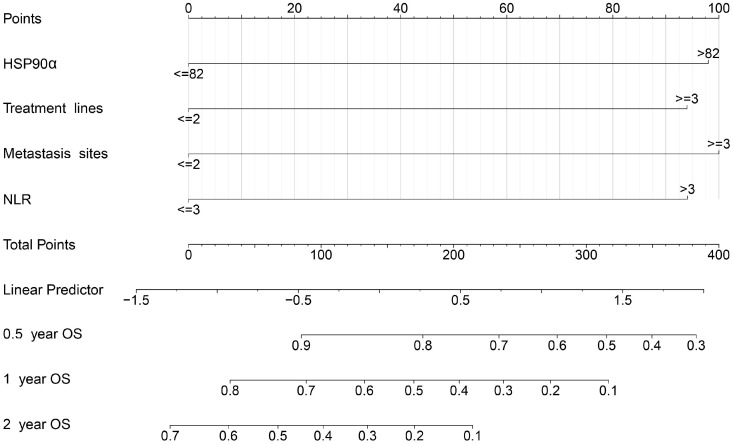
Nomogram predicting of 0.5-, 1-, and 2-year OS rate in patients with advanced gastric cancer treated with PD-1 inhibitors. The value of each variable is associated with a score. The scores corresponding to the different variables are added together to calculate the subject's total score.

Based on independent prognostic indicators (plasma HSP90α, NLR, and clinical parameters) with P<0.05 in the Cox multifactorial analysis, a nomogram was created. A total score was calculated by adding the values for each variable, and this score was then shown on an overall scale (Figure 3). Additionally, the nomogram graphically forecasted the research cohort's 0.5-, 1-, and 2-year OS rates for patients.

**Figure 4:**
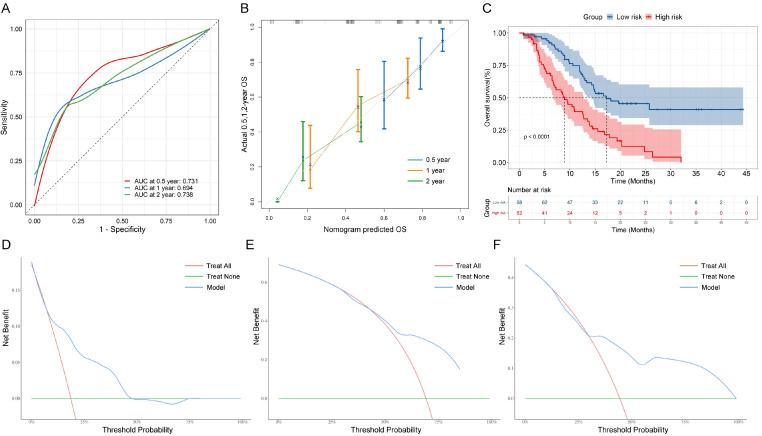
Performance of the nomogram. (A) Receiver operating characteristic curve (ROC) curves. The ability of the nomograms at 0.5-, 1-, and 2-year was measured according to the area under the curve (AUC) values for the cohort. (B) Calibration curves of the nomograms between predicted and observed 0.5-, 1-, and 2-year OS of AGC patients. (C) Kaplan-Meier curves of low-risk and high-risk of AGC patients based on OS nomograms predictions. DCA curves of OS prediction of the nomogram. (D) 0.5-year overall survival (OS). (E) 1-year overall survival. (F) 2-year overall survival.

After using nomograms for risk prediction, it is necessary to validate their accuracy and test their efficacy through indicators. Concretely, ROC curves were used to evaluate the sensitivity and specificity of nomograms. The AUC values for OS rates at 0.5, 1, and 2-year were 0.731, 0.694, and 0.738, respectively (Figure 4A), which demonstrated a good predictive value. In addition, Figure 4B shows calibration curves, which closely followed the 45-degree diagonal, indicating a high degree of consistency between the model's anticipated risk probabilities and the actual observation. Besides, DCA curves show that the nomogram model shows better clinical benefits at different thresholds (Figure 4D-4F). Finally, based on the overall prognostic risk score, we categorized patients into 2 subgroups: low- and high-risk group. Patients in the high-risk group had a considerably shorter OS than those in the low-risk group, according to the Kaplan-Meier curve and Log-rank test (P<0.001, Figure 4C).

### Subgroup survival analysis

**Figure 5:**
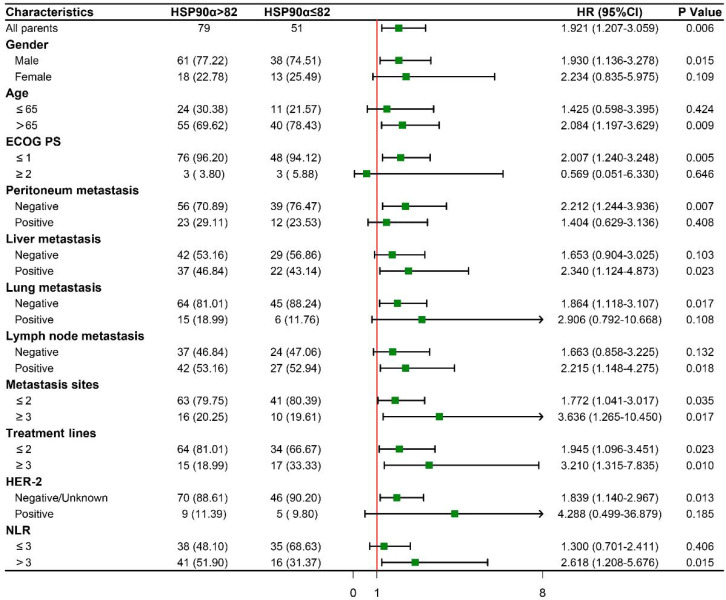
Subgroup survival analysis based on baseline clinical characteristics.

To further evaluate the prognostic significance of HSP90α in subgroups with various clinical characteristics. We drew forest plots based on Cox regression analysis to explore its prognostic value (Figure 5). Male patients with high HSP90α had a greater risk of OS than those with low HSP90α levels (HR: 1.930, 95% CI: 1.136-3.278, P=0.015), whereas there was no difference in female patients. And in the advanced age and low ECOG PS subgroup, compared to the low HSP90α group, the prognosis for the high HSP90α group was much poorer (HR: 2.084, 95% CI: 1.197-3.629, P=0.009; HR: 2.007, 95% CI: 1.240-3.248, P=0.005). Regarding metastasis sites, there was a significant difference between the high and low HSP90α groups in the peritoneal metastasis-negative, liver metastasis-positive, lung metastasis-negative, and lymph node metastasis-positive groups, respectively (HR: 2.212, 95% CI: 1.244-3.936, P=0.007; HR: 2.340, 95% CI: 1.124-4.873, P=0.023; HR: 1.864, 95% CI: 1.118-3.107, P=0.017; HR: 2.215, 95% CI: 1.148-4.275, P=0.018). Notably, high HSP90α level was an adverse prognostic factor in each subgroup regardless of the number of metastasis sites and different treatment lines. (metastasis sites≤2: HR: 1.772, 95% CI: 1.041-3.017, P=0.035; metastasis sites≥3: HR: 3.636, 95% CI: 1.265-10.450, P=0.017; treatment lines≤2: HR: 1.945, 95% CI: 1.096-3.451, P=0.023; treatment lines≥3: HR: 3.210, 95% CI: 1.315-7.835, P=0.010). Whereas in non-HER-2 positive and NLR>3 patients with high levels of HSP90α had a worse prognosis (HR: 1.839, 95% CI: 1.140-2.967, P=0.013; HR: 2.618, 95% CI: 1.208-5.676, P=0.015).

## Discussion

Many studies have shown that HSP90α released by cancer cells promotes cancer cell migration, invasion, and angiogenesis 10,22. And the level of HSP90α in plasma strongly correlates with tumour growth and distant metastasis 23. For these reasons, the diagnostic and prognostic utility of plasma HSP90α has received widespread attention in many malignancies, including hepatocellular carcinoma 19, lung adenocarcinoma 24, and colorectal cancer 17. However, its prognostic value in cancer immunotherapy, especially GC, has rarely been reported. In this study, plasma HSP90α levels were significantly associated with the short-term efficacy of PD-1 inhibitor therapy, and elevated HSP90α levels indicated disease progression. Additionally, we integrated four independent prognostic factors—HSP90, NLR, treatment lines, and metastatic sites—to construct a novel and convenient nomogram model for assessing the prognosis of gastric cancer patients receiving PD-1 inhibitor therapy, which aids in identifying potential beneficiaries of immunotherapy. To our knowledge, this study is the first to report the value of plasma HSP90α in monitoring the efficacy of immunotherapy and assessing prognosis in gastric cancer.

Concretely, we examined the therapeutic utility of plasma HSP90α in 130 AGC patients receiving PD-1 inhibitor treatment and found that the short-term effectiveness of GC patients was strongly correlated with dynamic changes in plasma HSP90α. After controlling for potential bias by PSM, we found that baseline plasma HSP90α was an independent prognostic predictor for AGC patients receiving PD-1 inhibitors treatment. Additionally, we constructed the nomogram according to four independent prognostic factors obtained, which were used to differentiate patients' clinical prognosis better. Finally, we further characterized the prognostic value of plasma HSP90α in various subgroups by grouping them according to different clinical characteristics. To our knowledge, this research firstly reports the value of HSP90α in efficacy monitoring and prognostic assessment in GC immunotherapy, which is of great importance.

Concerning baseline characteristics, plasma HSP90α was linked to a high level of NLR in AGC patients (Table 1). It is well known that NLR can reflect the systemic inflammatory burden of patients and is widely used in the prognostic evaluation of cancer 25. Recently, NLR has been reported to be a marker reflecting the effect of AGC treated with PD-1 inhibitors and served as an independent prognostic factor 26,27. This suggests that plasma HSP90α levels and NLR are correlated and may be associated with the prognosis of AGC patients treated with PD-1 inhibitors.

While HSP90α alterations could be a reflection of how well a patient is responding to therapy 28, only a small number of studies have examined how PD-1 inhibitors affect HSP90α dynamically. Our research shows that in AGC patients receiving PD-1 inhibitors, HSP90α is highly correlated with short-term results. Concretely, HSP90α decreased significantly in patients who obtained PR and remained stable in patients whose efficacy was assessed as SD status. However, HSP90α levels elevated significantly during short-term disease progression (Figure 2), consistent with our previous reports on malignant melanoma 29. These results revealed that HSP90α might be a crucial marker for reflecting tumour burden and monitoring the short-term efficacy in immunotherapy for AGC patients. In addition, our study has clarified that when PD-1 inhibitor treatment ultimately failed, patients showed significantly elevated levels of HSP90α compared to baseline (Figure 2). To our knowledge, this is the first report on plasma HSP90α in assessing the short-term efficacy of PD-1 inhibitors.

Plasma HSP90α has shown favourable value in diagnosing disease, monitoring recurrence, and evaluating prognosis in breast cancer 14, lung cancer 15, and pan-cancers 30. However, little is known about the value of plasma HSP90α in PD-1 inhibitor treatment when it comes to immunotherapy. A recent large multicenter study showed that HSP90α is a prognostic marker for hepatocellular cancer. Interestingly, in the subgroup analyses of the ICIs combined targeted treatment and the ICIs combined transcatheter arterial chemoembolization (TACE), OS was better in the low HSP90α group than in the high HSP90α group 19. Moreover, in a retrospective study that included 136 advanced lung cancer patients receiving a PD-1/PD-L1 inhibitor combined chemotherapy, high plasma HSP90α was an independent prognostic predictor for poor PFS and OS 31. Similarly, in our study, AGC patients treated with PD-1 inhibitors who had lower plasma HSP90α levels before and after PSM had significantly longer OS than those with higher plasma HSP90α. (Table 2, Table 3). However, a recent study focused on the significance of HSP90α in diagnosing GC 32. Despite having a modest diagnostic utility in GC, plasma HSP90α has not been found to have a substantial prognostic value. The reasons for this discrepant result are multiple. Firstly, there were differences in the target populations of the two studies. Second, the division of HSP90α is based on the results of the ROC curve, focusing on exploring its diagnostic value, and may not be the optimal cutoff value for prognostic grouping. Finally, the authors did not consider the effects of covariates and confounding factors in their prognostic analysis, which ultimately yielded negative results.

In our research, metastatic sites, treatment lines, and NLR level were also independent prognostic predictors in AGC patients treated with PD-1 inhibitors (Table 2). It is well known that patients with various numbers of treatment lines have significantly varying prognoses 33. Besides, the number of metastatic sites reflects the tumour burden of tumour patients, and previous research has reported that metastatic sites are an independent prognostic predictor for immunotherapy in GC 34. And NLR was a poor predictive factor in AGC patients treated with PD-1 inhibitors in a retrospective analysis, consistent with our findings 35,36.

Based on the four independent prognostic factors mentioned above, we developed and validated the nomogram for predicting the prognosis of AGC patients treated with PD-1 inhibitors (Figure 3). And the model showed great predictive performance, acceptable discrimination and calibration (Figure 4). This is similar to the results of a previous study that constructed a nomogram for gastric cancer immunotherapy based on inflammatory markers and clinical characteristics 37, and is superior to the relevant predictive models of traditional chemotherapy 38. Notably, our study uniquely emphasized the importance of plasma HSP90α, which has not received attention in previous studies. Besides, markers included in the model were readily available at baseline, thus providing important survival information for patients categorized by nomograms, and may help to identify patients who could benefit from immunotherapy, thereby rationalizing treatment strategies. Obviously, compared with traditional methods, the nomogram we developed is clinically important for PD-1 inhibitors of AGC, which helps to identify the potential benefit population of immunotherapy and helps physicians' clinical decision-making. In sum, our study extends previous observations by demonstrating a similar association between HSP90α and the prognosis of AGC received PD-1 inhibitors treatment and is the first to establish the prognostic nomogram model.

After that, we further explored the prognostic value of HSP90α in different subgroups (Figure 5). In the male, advanced age, and low ECOG PS subgroups, the prognosis of the high HSP90α group was considerably poorer than that of the low HSP90α group. Although the prognostic value of HSP90α varied somewhat among patients with different subgroups, high HSP90α levels were a poor prognostic factor in every subgroup, regardless of AGC patients' metastatic sites and treatment lines. Similar results were also in patients in the non-HER-2 positive and NLR>3 groups. This may be related to the heterogeneity of efficacy in different populations and the limited sample size, and future large-scale prospective studies are needed for further justification.

The mechanism of HSP90α in the therapy with PD-1 inhibitors is currently not fully understood. Most investigations have demonstrated that cancer patients, particularly those with advanced disease, have significantly higher plasma HSP90α than healthy individuals 39. And the ability of tumour cells to infiltrate, migrate, and form tumours in mice is due to the secreted form of HSP90α. The ability of tumour cells to move, invade, and metastasize is eliminated by CRISPR/Case9 knocking down HSP90α 22. Previous studies have found that HSP90 inhibitors potently reduce the expression of PD-L1 through regulating of STAT-3 and c-Myc. Interestingly, HSP90 inhibitors also influence how other checkpoint proteins, like PD-L2, are expressed on their surface. HSP90 inhibitors dramatically decreased the surface expression of PD-L1 on tumour cells in the MC-38 synthetic murine tumour model 40. Additionally, HSP90 inhibitors stimulated the quantity of activated CD8+ T cells in the tumour microenvironment and boosted T-cell-mediated anti-tumour immune responses 41. These findings provide further rationale for using HSP90 inhibitors combined with immunotherapy for cancer treatment. Encouragingly, results from a recent phase Ib trial of oral HSP90 inhibitor TAS-116 showed a manageable safety profile and anti-tumour activity combined with nivolumab for treating colorectal cancer and other solid tumours 42. In summary, HSP90 inhibitors can reduce PD-L1/L2 expression on tumour cells, removing T cell suppression. And the cytotoxic effector capabilities of the T cells can be resumed, thus killing tumour cells 43. Further researches are required to investigate the application of HSP90 inhibitors in cancer immunotherapy in the future.

To our knowledge, this is the first report on the efficacy monitoring and prognostic evaluation of plasma HSP90α for the PD-1 inhibitors treatment of AGC patients. However, The present study does have certain restrictions. Firstly, this study was single-center retrospective research with limited samples, and the generalizability of our findings will also need to be confirmed by external validation in a variety of groups. Secondly, due to the limited sample size, clinicopathological characteristics such as PD-L1 expression, MMR/MSI status, and types of PD-1 inhibitor were not included in this study for tumour classification. Finally, due to the limited sample size some potential unknown biases may affect our results. Therefore, a larger multicenter and prospective study is needed to verify our outcomes.

## Conclusion

In summary, the current research demonstrated that plasma HSP90α was significantly associated with NLR level in AGC patients at baseline. Additionally, decreased levels of HSP90α were associated to better short-term efficacy of PD-1 inhibitor treatment, whereas elevated levels of HSP90α suggested disease progression. Notably, plasma HSP90α, treatment lines, metastatic sites, and NLR level were independent prognostic predictors for OS in AGC patients treated with PD-1 inhibitors before and after PSM. Using these four independent prognostic factors, we contributed nomogram for evaluating the prognosis of AGC patients treated with PD-1 inhibitors, which can help to identify potential beneficiary populations of immunotherapy.

## Figures and Tables

**Table 1:** Baseline characteristics of AGC patient treated with PD-1 inhibitors

Characteristics	HSP90α (Before PSM)		P value^2^	HSP90α (After PSM)		P value^2^
	≤82, N=51^1^	>82, N=79^1^		≤82, N=41^1^	>82, N=41^1^	
Gender			0.724			0.557
Male	38 (75%)	61 (77%)		33 (80%)	35 (85%)	
Female	13 (25%)	18 (23%)		8 (20%)	6 (15%)	
**Age**			0.986			0.594
≤65	33 (65%)	51 (65%)		31 (76%)	33 (80%)	
>65	18 (35%)	28 (35%)		10 (24%)	8 (20%)	
**ECOG PS**			0.679			>0.999
≤1	48 (94%)	76 (96%)		40 (98%)	39 (95%)	
≥2	3 (6%)	3 (4%)		1 (2%)	2 (5%)	
**Peritoneum metastasis**			0.483			0.607
Negative	39 (76%)	56 (71%)		30 (73%)	32 (78%)	
Positive	12 (24%)	23 (29%)		11 (27%)	9 (22%)	
**Liver metastasis**			0.679			0.507
Negative	29 (57%)	42 (53%)		23 (56%)	20 (49%)	
Positive	22 (43%)	37 (47%)		18 (44%)	21 (51%)	
**Lung metastasis**			0.275			>0.999
Negative	45 (88%)	64 (81%)		35 (85%)	35 (85%)	
Positive	6 (12%)	15 (19%)		6 (15%)	6 (15%)	
**Lymph node metastasis**			0.980			0.825
Negative	24 (47%)	37 (47%)		19 (46%)	20 (49%)	
Positive	27 (53%)	42 (53%)		22 (54%)	21 (51%)	
**Metastasis sites**			0.928			0.432
≤2	41 (80%)	63 (80%)		33 (80%)	30 (73%)	
≥3	10 (20%)	16 (20%)		8 (20%)	11 (27%)	
**Treatment lines**			0.064			0.775
≤2	34 (67%)	64 (81%)		33 (80%)	34 (83%)	
≥3	17 (33%)	15 (19%)		8 (20%)	7 (17%)	
**Treatment types**			0.783			0.416
Chemotherapy + targeted therapy	16 (31%)	21 (26%)		12 (29%)	10 (24%)	
Chemotherapy	30 (59%)	48 (61%)		27 (66%)	25 (61%)	
Others	5 (10%)	10 (13%)		2 (5%)	6 (15%)	
**HER-2**			0.775			>0.999
Negative/Unknown	46 (90%)	70 (89%)		37 (90%)	36 (88%)	
Positive	5 (10%)	9 (11%)		4 (10%)	5 (12%)	
**NLR**			**0.021**			0.822
≤3	35 (69%)	38 (48%)		25 (61%)	24 (59%)	
>3	16 (31%)	41 (52%)		16 (39%)	17 (41%)	

^1^N(%) ^2^Pearson's chi-squared test; Fisher's exact test

**Table 2:** Cox regression analysis of OS in patients with AGC treated with PD-1 inhibitors before propensity score matching

	Univariate		Multivariate	
	HR^1^ (95% CI)^1^	P value	HR^1^ (95% CI)^1^	P value
**Gender**				
Male	1 (Reference)			
Female	1.166 (0.689-1.972)	0.567	-	-
**Age**				
≤65	1 (Reference)			
>65	0.830 (0.520-1.323)	0.433	-	-
**ECOG PS**				
≤1	1 (Reference)			
≥2	0.945 (0.345-2.586)	0.912	-	-
**Peritoneum metastasis**				
Negative	1 (Reference)			
Positive	1.782 (1.127-2.816)	0.012	1.183 (0.709-1.974)	0.519
**Liver metastasis**				
Negative	1 (Reference)			
Positive	0.812 (0.526-1.255)	0.348	-	-
**Lung metastasis**				
Negative	1 (Reference)			
Positive	1.220 (0.707-2.106)	0.475	-	-
**Lymph node metastasis**				
Negative	1 (Reference)			
Positive	0.999 (0.649-1.538)	0.997	-	-
**Metastasis sites**				
≤2	1 (Reference)			
≥3	2.182 (1.307-3.644)	0.002	2.262 (1.327-3.856)	**0.003**
**Treatment lines**				
≤2	1 (Reference)			
≥3	1.585 (0.976-2.574)	0.060	2.158 (1.290-3.610)	**0.003**
**Treatment types**		0.124		
Chemotherapy+ targeted therapy	1 (Reference)			
Chemotherapy	0.826 (0.504-1.354)	0.449	-	-
Others	1.636 (0.800-3.344)	0.177	-	-
**HSP90α**				
≤82	1 (Reference)			
>82	1.921 (1.207-3.059)	0.005	2.229 (1.370-3.627)	**0.001**
**HER-2**				
Negative/ Unknown	1 (Reference)			
Positive	0.462 (0.201-1.063)	0.063	0.545 (0.234-1.266)	0.158
**NLR**				
≤3	1 (Reference)			
>3	2.092 (1.357-3.225)	0.001	2.159 (1.383-3.369)	**0.001**

^1^HR = Hazard Ratio, CI = Confidence Interval

**Table 3:** Cox regression analysis of OS in patients with AGC treated with PD-1 inhibitors after propensity score matching

	Univariate		Multivariate	
	HR^1^ (95% CI)^1^	P value	HR^1^ (95% CI)^1^	P value
**Gender**				
Male	1 (Reference)			
Female	1.040 (0.484-2.235)	0.919	-	-
**Age**				
≤65	1 (Reference)			
>65	0.689 (0.370-1.285)	0.239	-	-
**ECOG PS**				
≤1	1 (Reference)			
≥2	2.092 (0.646-6.772)	0.208	-	-
**Peritoneum metastasis**				
Negative	1 (Reference)			
Positive	2.180 (1.176-4.043)	0.011	1.484 (0.724-3.043)	0.281
**Liver metastasis**				
Negative	1 (Reference)			
Positive	0.811 (0.461-1.427)	0.468	-	-
**Lung metastasis**				
Negative	1 (Reference)			
Positive	0.897 (0.420-1.918)	0.779	-	-
**Lymph node metastasis**				
Negative	1 (Reference)			
Positive	1.273 (0.724-2.238)	0.402	-	-
**Metastasis sites**				
≤2	1 (Reference)			
≥3	2.863 (1.549-5.293)	<0.001	2.978 (1.587-5.587)	**0.001**
**Treatment lines**				
≤2	1 (Reference)			
≥3	1.691 (0.881-3.247)	0.110	2.010 (1.019-3.963)	**0.044**
**Treatment types**		0.319		
Chemotherapy+targeted therapy	1 (Reference)			
Chemotherapy	0.858 (0.446-1.647)	0.644	-	-
Others	1.689 (0.640-4.459)	0.290	-	-
**HSP90α**				
≤82	1 (Reference)			
>82	1.704 (0.967-3.004)	0.062	1.977 (1.099-3.559)	**0.023**
**HER-2**				
Negative/Unknown	1 (Reference)			
Positive	0.501 (0.180-1.394)	0.177	0.565 (0.201-1.590)	0.280
**NLR**				
≤3	1 (Reference)			
>3	1.762 (1.000-3.105)	0.047	1.888 (1.061-3.358)	**0.031**

^1^HR = Hazard Ratio, CI = Confidence Interval
